# Corneal blindness in the developing world: The role of prevention strategies

**DOI:** 10.12688/f1000research.141037.2

**Published:** 2024-03-05

**Authors:** Anahita Kate, Sayan Basu

**Affiliations:** 1 Shantilal Shanghvi Cornea Institute, LV Prasad Eye Institute, Vijayawada, Andhra Pradesh, India; 2 Centre for Ocular Regeneration (CORE), Prof. Brien Holden Eye Research Centre, Champalimaud Translational Centre for Eye Research, LV Prasad Eye Institute, Hyderabad, Telangana, India; 3 Shantilal Shanghvi Cornea Institute, LV Prasad Eye Institute, Hyderabad, Telangana, India

**Keywords:** corneal blindness, corneal transplantation, keratoplasty, eye banking, prevention of blindness

## Abstract

Corneal blindness is an important contributor to the burden of global blindness and has a greater prevalence in low-income countries of the developing world where resources and infrastructure are limited. The causes of corneal blindness too are different from high-income countries and include infectious keratitis, ocular trauma, and xerophthalmia. Persons with these indications tend to have unfavourable outcomes after corneal transplantation, limiting their chances of benefitting from this sight-saving procedure. However, most causes of corneal blindness in the developing world are preventable. This highlights the importance of understanding the unique challenges in these regions and the need for targeted interventions. This article discusses various prevention strategies, including primordial, primary, and secondary prevention, aimed at reducing the burden of corneal blindness in low-income countries. These include capacity building, training, and awareness campaigns to reduce the risk factors of ocular trauma, infectious keratitis, and to improve access to first aid. It is also important to promote safe eye practices and tackle nutritional deficiencies through public health interventions and policy changes. Providing the required training to general ophthalmologists in the management of basic corneal surgeries and diseases and enhancing the accessibility of eye care services in rural areas will ensure early treatment and prevent sequelae. Current treatment modalities belong to the tertiary level of prevention and are largely limited to corneal transplantation. In developing nations, there is a scarcity of donor corneal tissue necessitating an urgent expansion of eye banking services. Alternative approaches to corneal transplantation such as 3D printed corneas, cultured stem cells, and biomaterials should also be explored to meet this demand. Thus, there is a need for collaborative efforts between healthcare professionals, policymakers, and communities to implement effective prevention strategies and reduce the prevalence of corneal blindness in the developing world.

## Introduction

The faculty of sight is one of our most essential senses and is necessary for basic survival. This is reflected not only in the abundance of published literature on vision compared to other senses but also in the perception of sight as the most valued sense by the general public. ^1^^,^^2^ The plethora of policies and programmes targeted at reducing the burden of blindness and its associated morbidity also stem from this idea. The causes of blindness targeted within these schemes are identified through various epidemiological studies or population based surveys and include etiologies such as cataract, uncorrected refractive error, glaucoma and age related macular degeneration. ^3^^,^^4^ Interventions such as dispensing glasses, improving access to cataract surgeries and glaucoma/retina screening camps go a long way in decreasing the disease burden. Although this approach is practical on a global scale, it may inadvertently overlook certain diseases which are important causes of visual impairment in certain populations.

Blindness secondary to corneal pathologies is one such category that affects nearly 5.5 million individuals bilaterally and another 6 million people unilaterally. ^5^ ^(p198)^ A variety of causes including poor nutrition at birth, infected ocular abrasions from agricultural work, or workplace injuries due to lack of protective glasses lead to corneal blindness. The majority of these cases are concentrated in developing countries where limited resources and inadequate infrastructure pose significant obstacles to addressing this problem effectively. Corneal blindness presents unique challenges as its treatment modalities are not as straightforward or universally effective as cataract surgery. This article will discuss the unique nature of the causes of corneal blindness in developing countries and explore various strategies that can be implemented to reduce the burden and impact of this condition.

## Blindness and the relative contribution of corneal blindness

The global crude prevalence of vision loss is 14% of which moderate-severe vision impairment has a prevalence of 3.7%, while blindness is 0.5%. ^6^ There is a disparity within the distribution of these cases which is highlighted by the Global Vision Database (GVD) and its regional classification system that classifies regions based on geographic and epidemiological similarity. The age-adjusted prevalence of vision loss is 4.6% in the “high-income region” which increases to 18.2% in “Sub-Saharan Africa” and is the highest in “South Asia” (22.2%). ^6^ A similar trend is seen in the age-adjusted prevalence of blindness with values of 0.2%, 1% and 0.9% in the high-income region, Sub-Saharan Africa, and South Asia respectively. ^6^ This difference is present not only in the magnitude of visual morbidity but also the causative pathologies. The major diseases responsible for blindness as per the Global Burden of Disease Study in the “high income region” are glaucoma (28.2%) and age-related macular degeneration (ARMD) (21.6%). ^3^^,^^6^ However, these diseases contribute lesser to the overall cases of blindness in “South Asia” with 6.4% and 3% of blindness being secondary to glaucoma and ARMD respectively. ^4^ Cataract is the most common cause of blindness in this region (63.1%). Interestingly, the second highest pathology in South Asia (16.8%) is a broad category of “residual causes”, and this is where corneal blindness resides. Individual studies from countries in this region will help understand the proportion of blindness due to corneal pathologies and together, these cases account for a majority of the global burden of corneal blindness. ^7^^–^^11^ This difference pertains not only to the absolute numbers of individuals affected by corneal blindness, but also extends to the age distribution of the affected population. ^5^ The incidence of childhood corneal vision loss in developing countries is nearly 20 times greater than that of developed ones. ^12^ The associated effect on the quality of life will be greater in this subset of the population and will directly impact their socio-economic productivity. The causes of corneal blindness (see below) and its etiology may vary between low- and high-income settings. Therefore, national programmes in developing countries that focus on cataract or refractive errors may be inadequate to tackle other conditions that cause visual morbidity. A full-fledged programme will need focused, population-based studies to identify the full range of conditions contributing to severe vision loss and blindness. The availability of robust data around corneal blindness in particular, will enable the development of comprehensive programs designed to address its challenges at both regional and nationwide scales.

## Causes of corneal blindness

Establishing the true incidence and prevalence of corneal blindness is a challenge because most of the existing literature reports hospital-based rates or uses surrogate measures such as indications for transplantation. The latter may not be representative as there are several eyes with corneal blindness which may not be amenable to corneal transplantation. Furthermore, lack of access to transplantation in developing countries may result in underestimation of the prevalence of the indications in these areas. Within the developed countries, keratoplasties are most commonly carried out for keratoconus, pseudophakic or aphakic bullous keratopathy and re-grafts. ^7^^,^^13^^–^^15^ This is in contrast to developing countries where corneal scars, ocular trauma, and infectious keratitis are the most common indications for a corneal graft. ^7^^,^^13^^,^^16^^,^^17^

The importance of this difference in indications is reflected in the stark difference in the survival rates of grafts with the latter group having a poorer prognosis. ^13^^,^^18^^–^^20^ This occurs because of factors such as host bed vascularization, prior perforation, peripheral anterior synechiae and other chamber abnormalities that ensues during the course of the contributory disease. Another important difference in the pattern of indications is that a large proportion of the diseases in the developing countries are preventable. More than 80% of this visual morbidity is avoidable and this has to be considered in the context of the fact that these cases account for that of more than half of the world’s corneal blindness cases. ^9^^,^^16^ A similar pattern is observed in pediatric corneal blindness, where vitamin A deficiency, a preventable condition, emerges as the leading cause. ^21^^,^^22^ Furthermore, greater than 45% of the affected children reside in South Asian countries which highlights the need to reduce this prevalence and address the issue effectively. ^21^^,^^23^^,^^24^

## Measures to decrease the burden of corneal blindness

**Figure 1.**
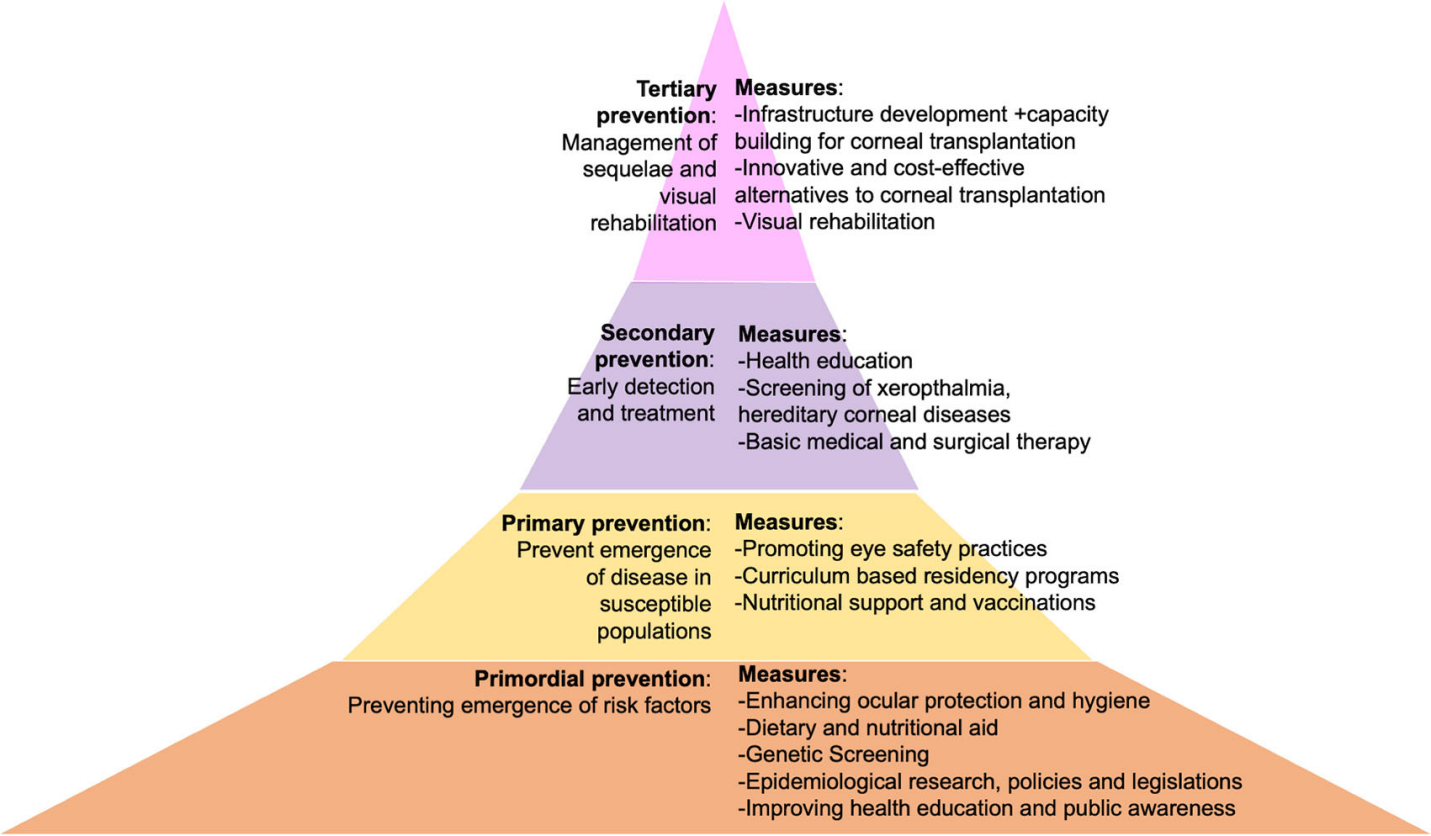
Figure depicting the four levels of prevention in the context of corneal blindness along with specific measures that can be implemented within each level. The lower two levels are broad indicating the widely available care provided, ensuring accessibility across different areas. The upper two levels are taller emphasizing the comprehensive nature and extensive range of services provided at those levels.

Corneal blindness has an adverse impact on daily functioning, social well-being and the overall quality of life. Thus, it is imperative to reduce its prevalence. Classifying these measures based on the level of prevention achieved will help understand where the lacunae lie, which can then be addressed. Briefly, these levels include primordial, primary, secondary, and tertiary prevention ( Figure 1). ^25^^,^^26^ Primordial prevention aims at preventing the emergence of risk factors in the general population for the disease being targeted. ^26^^,^^27^ Decreasing the incidence of a disease in a cohort with risk factors for the same is known as primary prevention, while screening and early detection of the established disease is secondary prevention. ^25^^–^^27^ Tertiary prevention is carried out to reduce the sequelae and to treat the complications that ensue from a given disease. ^25^^–^^27^

**Figure 2.**
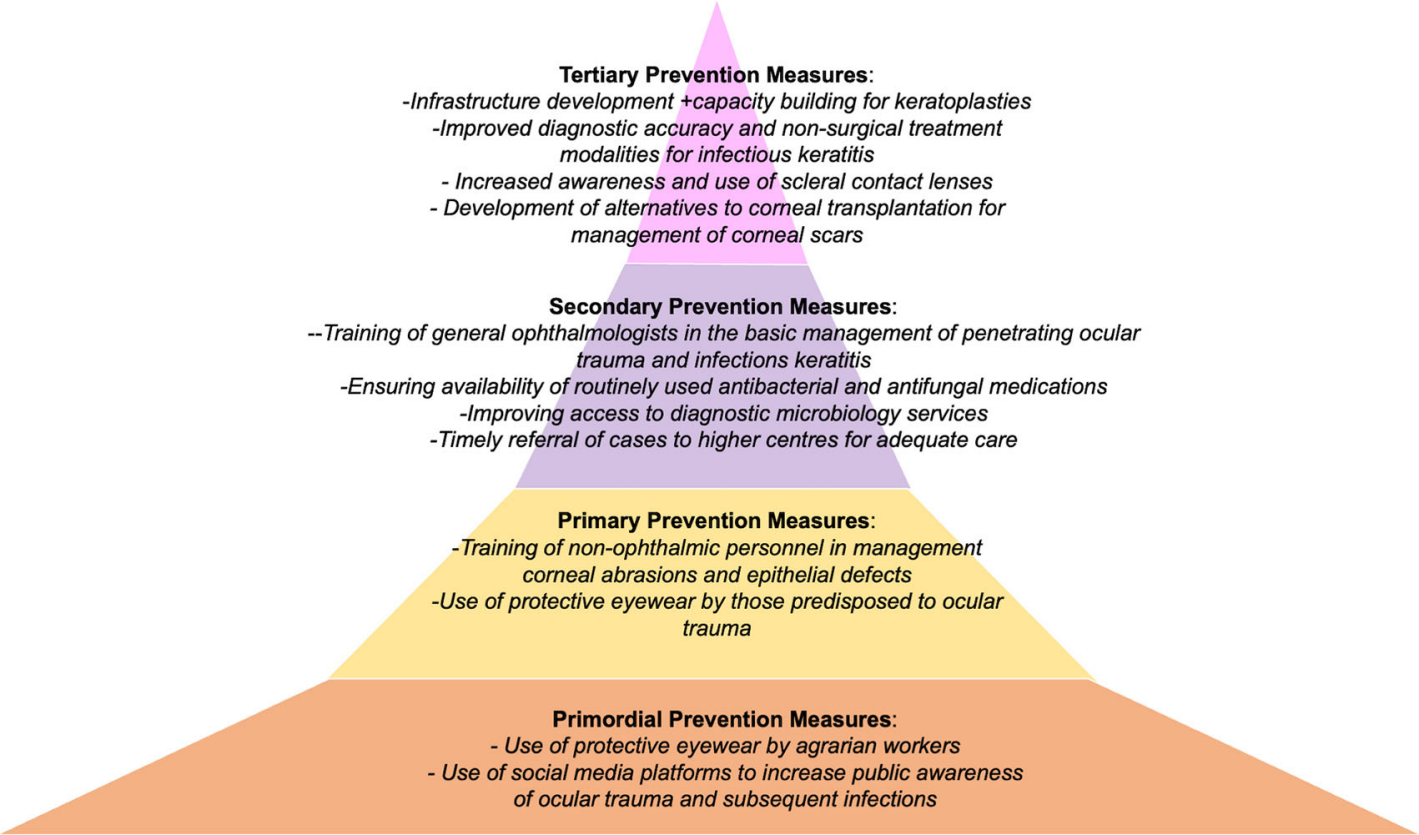
Figure depicting the four levels of prevention of corneal blindness due to corneal scar after ocular trauma (penetrating ocular trauma or infectious keratitis), with measures that can be implemented within each level.

**Figure 3.**
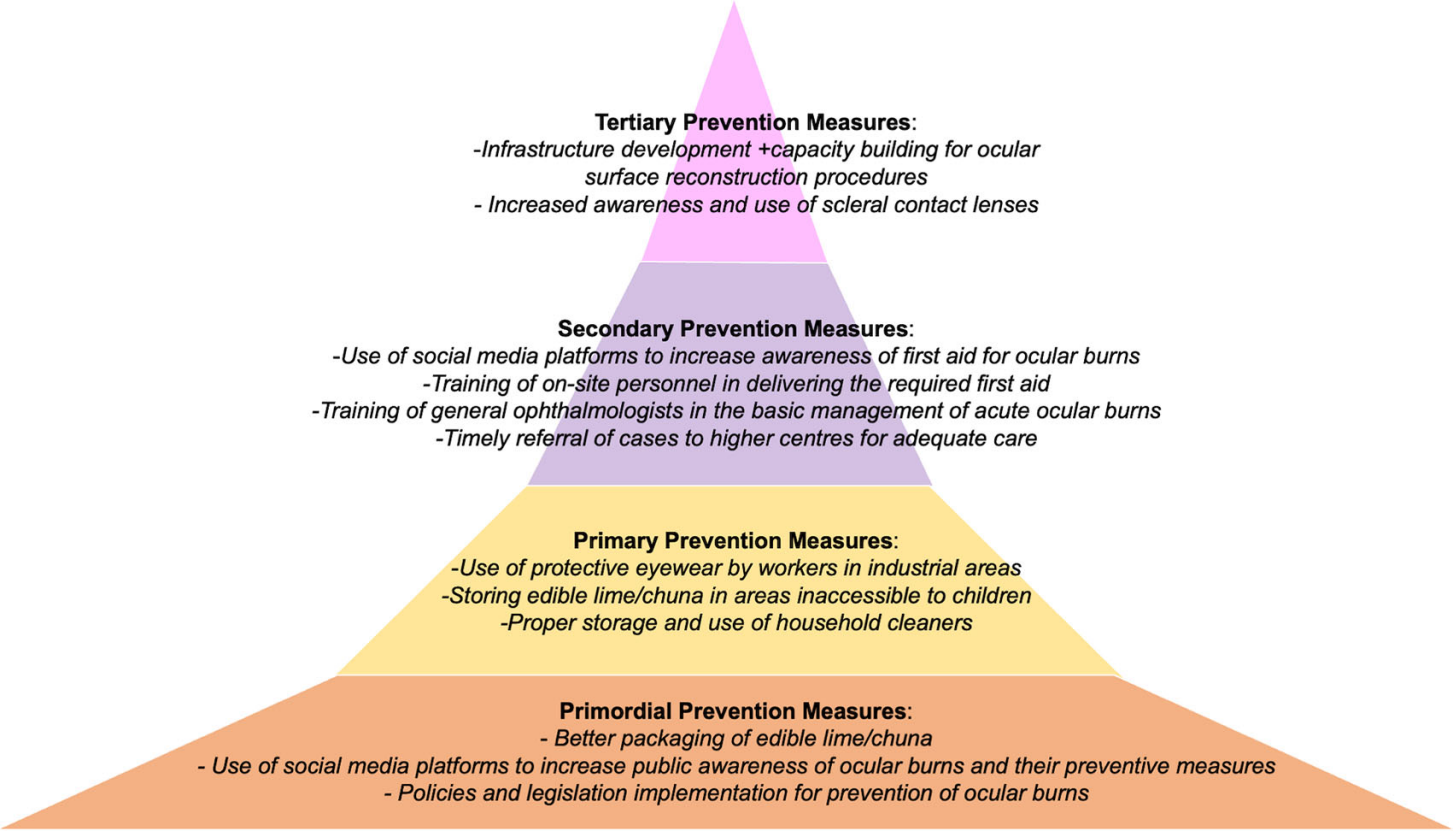
Figure depicting the four levels of prevention of corneal blindness due to acute ocular burns, with measures that can be implemented within each level.

Corneal transplantation, which is the mainstay of treatment of corneal blindness, falls under tertiary prevention of corneal blindness. While eye banking and corneal transplantation are indispensable tools for any corneal surgeon, relying solely on these interventions are insufficient to eradicate the problem of corneal blindness in the developing countries. This is because the indications differ significantly in these regions, resulting in grafts in developing countries having an unfavourable prognosis. Moreover, the majority of the corneal blindness in these regions arise from preventable causes. This is an important distinction to acknowledge because currently there is a dearth of eye banking services and infrastructure, and several agencies and policies are aimed at eliminating this deficiency. While ramping up this sector is crucial, an equal effort needs to be directed at the other end of the spectrum to reduce the incidence of corneal blindness itself by preventing the emergence of these diseases. Additionally, it will also reduce the socioeconomic burden on both the patients and healthcare systems. The subsequent section will discuss the various strategies at different levels of prevention in the context of some of the diseases that are commonly implicated in corneal blindness in developing countries. This includes infectious keratitis, ocular trauma including chemical injuries, pseudophakic bullous keratopathy, dystrophies, and vitamin A deficiency. ^28^^,^^29^ Examples of what different levels of prevention would look like in two case examples of corneal scar and ocular burns due to ocular trauma have been presented in Figures 2 and 3.

### Primordial prevention strategies

*Enhancing ocular protection and hygiene:*

Promoting eye safety practices such as wearing protective eyewear when engaging in activities that pose a risk of eye injury, such as sports and construction work, can prevent corneal injuries that may lead to blindness. Encouraging good hygiene practices such as regular handwashing, avoiding touching the eyes with dirty hands, and ensuring safe contact lens hygiene can help reduce the incidence of corneal infections. Social media platforms can effectively disseminate the message and reach a wide audience, amplifying the awareness and impact of these initiatives.

*Dietary and nutritional aid:*

Ensuring a well-balanced diet rich in essential nutrients, particularly vitamin A, can reduce the burden of vitamin A deficiency related corneal pathologies in children and young adults. This can be tackled by increasing awareness amongst healthcare workers who are in direct contact with preschool children and young women. These individuals can sensitize caregivers regarding the importance of a nutritious diet in preventing xerophthalmia. Additionally the use of fortified food can add to the daily intake of vitamin A. ^30^

*Genetic screening:*

Genetic screening for hereditary corneal dystrophies can identify individuals carrying recessive genes and help in premarital counselling. This will not only facilitate reducing the incidence of corneal blindness but also help identifying “at risk” groups who will benefit from screening of these diseases.

*Epidemiological research:*

Epidemiological research is one of the most significant gaps that requires immediate attention since this data will help improve our understanding of the risk factors, at-risk groups, and potential strategies to prevent corneal blindness. This is especially true with respect to establishing the true incidence and prevalence of corneal blindness which is currently calculated using surrogate measures such as indications of keratoplasty and hospital-based registries which are prone to their inherent biases. Identifying the most important factors responsible for these preventable causes of corneal blindness will allow concentrating efforts and resources on mitigating the same.

*Policies and legislations:*

With the data from large population-based studies, ophthalmic societies can propose and advocate legislations that promote eye safety and health. Examples include better packaging of edible lime which is an important cause of severe ocular burns in South Asia and ensuring standardised, appropriate protective eyewear for workers in factories and agrarian settings. ^31^ Implementing these policies and regulations at the national or community level can have a significant impact on primordial prevention.

*Improving health education and public awareness:*

Promoting programs that educate people on lifestyle modifications and health practices can decrease the risk of corneal diseases. This starts with increasing awareness of diseases such as infectious keratitis, vitamin A deficiency and ocular burns in the context of their consequences and measures of prevention. This can also involve engaging communities such as school employees and factory workers in these promotion activities and creating opportunities for active participation which can foster a sense of ownership and responsibility for eye health. Improving health care seeking practice in the event of any ocular trauma or suspicion of infection should be advocated. Additionally, procuring over the counter medications and usage of traditional remedies should be discouraged.

### Primary prevention

*Promoting eye safety practices:*

Promoting eye safety practices include wearing protective eyewear by workers in industries with a high-risk for ocular trauma, burns, and infections. Training should be provided to the workers to administer basic first aid at the work site. While there is some understanding of these predisposing factors and the individuals at risk, extensive research is required to comprehensively identify the exact risk factors associated with each condition and determine the populations most susceptible to it. This will also help advocate the necessary policy changes.

*Curriculum based residency programs:*

The implementation of well-defined guidelines for surgical training of residents and trainees will facilitate the evaluation of their performance and provide support whenever necessary. Adequate supervision and assistance based on each phase of the learning curve will help improve the outcomes of cataract surgery and decrease the prevalence of pseudophakic/aphakic bullous keratopathy.

*Nutritional support and vaccinations:*

Nutritional support and vaccinations that specifically target young children and pregnant women can help reduce the incidence of xerophthalmia related blindness which is an important and preventable cause of corneal blindness in low income countries. Current guidelines dictate that vitamin A prophylaxis is to be administered only to at-risk individuals. These doses are administered as per recommended protocols to pregnant women and children less than six years of age residing in areas which are endemic to vitamin A deficiency. ^22^ Similarly ensuring children are appropriately vaccinated against measles can help reduce the incidence of xerophthalmia. ^32^

### Secondary prevention

*Health education:*

Since the secondary prevention level is targeted at screening and early detection of the disease with prompt institution of treatment, specific measures will be unique to each disease. Furthermore, all measures will necessitate the presence of both trained personnel and ease of access to the same. The outcomes of ocular trauma, whether it be penetrating injuries or ocular burns, are significantly influenced by the promptness and effectiveness of the initial first aid/surgical interventions provided. Increased public awareness of these interventions will improve care seeking practice and permit setting up of first aid centres that can be manned by non-ophthalmic individuals.

*Screening and early detection:*

Screening programmes that target high-risk populations such as schools for vitamin A deficiency and predisposed groups for genetic corneal pathologies will help early detection of these diseases and enable timely interventions. Similarly setting up smaller units in villages with trained ophthalmic personnel can help screen and treat corneal epithelial defects and abrasions before they fulminate into infectious keratitis. ^33^^,^^34^

*Medical and surgical therapy:*

Ensuring the availability of medications targeting the microorganisms commonly involved in infectious keratitis in specific geographic regions is essential. Similarly setting up reliable and efficient labs that can detect these pathogens are also paramount to ensure an accurate microbiological diagnosis is being made. ^35^ Use of innovative techniques such as trypan blue for fungal filaments, that provide simple, rapid and reliable methods of examination will also enable early diagnosis of infectious keratitis. ^36^ Educating general ophthalmologists in triaging severity of infections and ocular trauma will enable appropriate and timely referrals to higher centres for the necessary care. Providing surgical training for fundamental procedures like corneal tear repairs, corneal foreign body removals, amniotic membrane grafts, and tarsorrhaphies, can not only ensure timely execution of these procedures but also alleviate the workload on advanced centres, thus, allowing them to concentrate on more complex cases.

### Tertiary prevention

This tier focuses on addressing the sequelae of different corneal pathologies and ensuring visual rehabilitation to improve the quality of life. The majority of the clinical care currently provided falls within this level, and this section will discuss existing approaches and propose methods to improve upon them.

*Corneal transplantation:*

This is the main modality of treatment for several corneal pathologies and aims to replace the diseased cornea with a healthy one. However, there are several issues that prevent optimal implementation of this procedure in developing countries.

The demand for donor corneas is not met by the supply and this needs to be addressed by improving the infrastructure and human resources. Estimates show that nearly 13 million individuals currently await a corneal transplantation globally and one million new cases get added every year. ^12^^,^^37^ This discrepancy between the demand and supply is exacerbated in developing countries. For example, In India, 100,000 transplantations per year are necessary to reduce the burden of corneal blindness. However, the procurement rate is less than 25% of this burden--and only half the harvested corneas are utilized. ^38^^–^^40^ Hence, to meet the needs of corneal transplantation, nearly 200,000 corneas need to be harvested. Ensuring these numbers requires a multipronged approach which targets the donor tissue availability as well as its storage and preservation. A multitiered system with eye banks that cater to a predefined target population can effectively expand their reach and secure an ample supply of donor corneas. This infrastructure should support not only traditional corneal transplantation but also the newer lamellar techniques. Increased public awareness of eye donation programmes and the need for donor corneas will help close the supply gap. The establishment of centralized registries, documenting patient lists and prioritizing their needs, can facilitate the allocation of this valuable resource to individuals in greatest need on a priority basis.

Capacity building plays a crucial role in facilitating the optimal utilization of corneal tissues. This includes not only the training of corneal surgeons in the fundamental aspects of keratoplasty but also paraclinical staff to ensure the accurate collection of viable donor corneas. Eye bank technicians need to be trained in preparing lamellar endothelial grafts. Ensuring the presence of trained corneal surgeons in rural areas will not only enhance access to keratoplasty facilities but also facilitate improved adherence to postoperative follow-up and care. ^41^ Providing training to corneal surgeons in lamellar keratoplasties and other emerging surgical modalities will aid in their widespread adoption and implementation. This will not only result in improved outcomes but also enable the utilization of multiple grafts from a single donor tissue, maximizing the potential benefits of each donated cornea. ^42^ Finally, corneal surgeons should be versed not only in surgical skills but also in judicious case selection which will go a long way in optimizing the utilization of this scarce resource and improving the outcomes of these grafts.

### Alternatives to corneal transplantation

The limitations of corneal transplantation have been discussed in the previous section which highlight how relying solely on keratoplasties is not a viable option for developing countries. Even for cases which have a good prognosis such as keratoconus or corneal opacifications without vascularization/anterior segment abnormalities, the postoperative course is subject to several issues which can affect the final visual outcome. This includes post-keratoplasty astigmatism, suture-related complications, graft infiltrates, and secondary glaucoma. ^43^^–^^45^ Moreover, the postoperative care demands rigorous adherence and relies heavily on the patient's diligent compliance, as well as the presence of a supportive socio-economic system. However, establishing such a system can often pose challenges, especially in developing countries. Hence, alternatives to keratoplasties that circumvent these issues and provide long-term effective and reliable outcomes are the need of the hour.

*Contact lenses (CL):*

Although CL have been around for several decades, it is with the advent of better lens materials and large scleral lenses with fluid reservoirs that they became game changers for corneal specialists. ^46^^–^^48^ They are indicated in a vast number of diseases that range from corneal scars and keratoconus to severe ocular surface diseases. ^49^^–^^52^ They not only improve the visual function by correcting the irregular astigmatism but also improve the overall health of the ocular surface. Enhancing awareness regarding the indications and benefits of these lenses will play a crucial role in promoting CL use. Training ophthalmologists and optometrists in dispensing these lenses will contribute to their broader distribution.

*Ocular surface reconstruction and artificial corneas:*

Very often patients with seemingly opaque corneas can experience complete visual rehabilitation through procedures such as limbal stem cell transplantation. Surgeries such as simple limbal epithelial transplantation can not only address the deficient limbal epithelial stem cells but also modulate the underlying corneal scar and decrease its density. ^53^^–^^56^ These procedures have stable long-term outcomes with good visual function, thus eliminating the need for keratoplasty in cases where corneal transplantation would not yield favourable outcomes. ^55^^,^^57^^,^^58^ Providing training to corneal surgeons in the indications, perioperative management, and surgical techniques related to these procedures will equip them with the necessary skills to effectively handle such cases. Dissemination of information on basic and complex ocular surface reconstruction procedures will allow continued medical education to occur. ^59^^–^^63^ Conducting periodic workshops will also help refresh these skills. Research endeavours are crucial to developing similar techniques that can circumvent the necessity for corneal grafting in these patients.

The use of prosthetic devices to replace the diseased cornea has been explored for several decades and recent advances have offered options that have good visual outcomes. ^64^^–^^66^ Furthermore, they do not require high grade corneal tissue, and thus help improve utilization rates. However, the retention and long-term efficacy of these devices remains a limitation. ^64^^–^^66^ With the advent of completely synthetic devices such as the CorNeat, corneal replacements can be carried out independent of donor corneal tissues. ^67^^,^^68^

*Molecular therapy:*

This is another appealing option that modulates scar formation during the process of corneal wound healing thus preventing the formation of the scar itself. Several interventions are being explored with respect to this and include bioactive molecules, exosomes, nanomedicine, recombinant viral vectors, and microRNAs. ^69^^,^^70^ However, these are still in the experimental phase and will require clinical validation prior to their adoption in routine clinical practice.

*Bioengineered cornea:*

Substitutes for the cornea are an attractive option aimed at addressing the issue of deficient donor tissue. These substitutes can be cell-based, which recreate individual layers of the epithelium, stroma and endothelium. ^71^ The other approach to tissue engineering is the scaffold-based models which are primarily for stromal regeneration. These models can be natural or synthetic. The former has a better capacity for integrating with the surrounding host tissue, while the latter can be modulated to the desired requirements. ^72^^,^^73^ Natural polymers can also be obtained from human donor corneas which are deemed unsuitable for transplantation. As a result, this approach enables optimal utilization of tissue resources and allows for the acquisition of multiple grafts from a single tissue source. ^72^ The use of 3D-printing technology to print an artificial cornea with raw materials such as collagen, alginate, and decellularized extracellular matrix is a promising solution to the problem of deficient donor corneas. ^74^

Other advances such as cultured endothelial cells which can be directly injected into the anterior chamber can not only be used as the primary treatment modality in pseudophakic bullous keratopathies but also for failed grafts. ^75^^,^^76^ This can provide a minimally invasive option to revive otherwise failed transplants. Other options include targeted gene therapy and anti-sense oligonucleotides. ^76^ Simple procedures such as removal of the diseased endothelium to pave the way for the surrounding endothelial cells in partial endothelial failure are also being explored. ^76^ These techniques that do not rely on donor tissue or specialized equipment are urgent requirements.

*Supportive care and rehabilitation:*

Persons with corneal blindness may suffer from a range of problems including but not limited to depression, economic and personal dependence, and mobility issues. Providing supportive care services and rehabilitation programs to these individuals to help them cope with the physical, emotional, and social aspects of their condition is a crucial aspect of tertiary care. This may involve physical therapy, occupational therapy, counselling, or support groups and helps them reintegrate into society. These services not only help maintain household independence but also financial independence to the greatest extent possible.

*Universal eye health care:*

The current standard of care can effectively treat a significant proportion of the causes of corneal blindness. However, there exist several barriers that prevent individuals afflicted with these diseases from accessing this care. In order to address these barriers, a comprehensive and collaborative approach is essential, involving various stakeholders including healthcare providers, policymakers, and both public and private financial aid organizations. ^77^ Incorporation of eye health into universal healthcare policies is imperative to ensure holistic care and this requires the necessary financial and human resource planning. By including eye care in these key areas, we can effectively address the diverse needs of individuals and communities and ensure accessible quality eye care services.

## Conclusion

The global distribution of corneal blindness exhibits significant disparities, predominantly affecting mid to low-income countries. Current treatment approaches primarily focus on the tertiary level of prevention but are often unable to guarantee long-term visual rehabilitation. Novel therapeutic options that are not only effective but also inexpensive and accessible are the need of the hour. Additionally, a substantial portion of corneal blindness cases stem from preventable causes. Redirecting efforts and allocating resources towards primordial and primary prevention would effectively alleviate the burden of corneal blindness. A collaborative and concerted effort from healthcare professionals and government entities, facilitated by effective policies, can succcessfully channel resources and focus on the prevention of corneal blindness. By preventing the disease itself, the resultant burden on the healthcare system will also reduce and the elimination of this form of blindness can lead to substantial positive economic outcomes. The societal benefits of improved quality of life and increased social integration will further contribute to building a world where everyone can live to their full potential.

## Data Availability

No data are associated with this article.
