# Supplementary material for: Corneal blindness in the developing world: The role of prevention strategies
Source: F1000Res. 2024 Mar 5;12:1309. Originally published 2023 Oct 11. [Version 2] doi: 10.12688/f1000research.141037.2 (PMC11009612; doi:10.12688/f1000research.141037.2)
Supplement: Peer review history

# Reviewer report: Reviewer response for version 2

**DOI:** 10.5256/f1000research.163206.r252914

**Published:** 2024-04-11

**Authors:** Martina Kropp

**Affiliations:** 1 University Hospitals of Geneva, Geneva, Switzerland; 2 Experimental Ophthalmology, University of Geneva, Geneva, Switzerland

The authors replied comprehensively to the comments of the reviews.

Is the topic of the opinion article discussed accurately in the context of the current literature?

Yes

Are arguments sufficiently supported by evidence from the published literature?

Yes

Are all factual statements correct and adequately supported by citations?

Yes

Are the conclusions drawn balanced and justified on the basis of the presented arguments?

Yes

Reviewer Expertise:

gene therapy, retina culture, neuroretinal degeneration, AMD, diabetic retinopathy, personalized medicine, regenerative medicine, iPSC, international ophthalmology

I confirm that I have read this submission and believe that I have an appropriate level of expertise to confirm that it is of an acceptable scientific standard.

# Reviewer report: Reviewer response for version 2

**DOI:** 10.5256/f1000research.163206.r252915

**Published:** 2024-03-18

**Authors:** Denise Loya Garcia

**Affiliations:** 1 Instituto de Ciencias Visuales y Oftalmología, Instituto de Oftalmologia Fundacion Conde de Valenciana IAP, Mexico City, Mexico City, Mexico

The authors have satisfactorily addressed the comments made by the reviewers.

 I have no further comments.

Is the topic of the opinion article discussed accurately in the context of the current literature?

Yes

Are arguments sufficiently supported by evidence from the published literature?

Yes

Are all factual statements correct and adequately supported by citations?

Yes

Are the conclusions drawn balanced and justified on the basis of the presented arguments?

Yes

Reviewer Expertise:

Cornea, External Diseases, Refractive Surgery.

I confirm that I have read this submission and believe that I have an appropriate level of expertise to confirm that it is of an acceptable scientific standard.

# Reviewer report: Reviewer response for version 1

**DOI:** 10.5256/f1000research.154448.r249004

**Published:** 2024-02-27

**Authors:** Martina Kropp

**Affiliations:** 1 University Hospitals of Geneva, Geneva, Switzerland; 2 Experimental Ophthalmology, University of Geneva, Geneva, Switzerland

The article summarizes current knowledge and state of the art prevention and treatment of corneal blindness. Authors state that current strategies in developing countries are insufficient and propose a 4-level prevention strategy to reduce prevalence of corneal blindness.

 The topic is of high importance and well addressed by the authors. They discuss the different reasons for corneal blindness in industrialized vs. developing countries pointing out that the different causes also need significantly different strategies to fight against it. A main difference is the fact that in developing countries many cases of corneal blindness are preventable and can thus, effectively avoided by mitigation strategies that are applied before patients become severely visually handicapped like eye protection with glasses or dietary vitamin A supplementation.

 The authors propose a comprehensive 4-level prevention strategy able to intervene at different stages of ocular care starting with primordial prevention (e.g., eye hygiene) to primary prevention (e.g., promoting eye safety practices) to secondary prevention (e.g., health education and screening programmes) to tertiary prevention (e.g., treatment infrastructure development). In the following, all options at all levels are mentioned and discussed including improvement of current care and potential future treatment approaches.

 The article is well written using an understandable style that is complete but compact. All factual statements and arguments are well justified by current literature. The only minor comment to make is that the text within the figures is quite small and it would be good, if the font size could be increased.

Is the topic of the opinion article discussed accurately in the context of the current literature?

Yes

Are arguments sufficiently supported by evidence from the published literature?

Yes

Are all factual statements correct and adequately supported by citations?

Yes

Are the conclusions drawn balanced and justified on the basis of the presented arguments?

Yes

Reviewer Expertise:

gene therapy, retina culture, neuroretinal degeneration, AMD, diabetic retinopathy, personalized medicine, regenerative medicine, iPSC, international ophthalmology

I confirm that I have read this submission and believe that I have an appropriate level of expertise to confirm that it is of an acceptable scientific standard, however I have significant reservations, as outlined above.

# Reviewer report: Reviewer response for version 1

**DOI:** 10.5256/f1000research.154448.r223571

**Published:** 2023-12-15

**Authors:** Denise Loya Garcia

**Affiliations:** 1 Instituto de Ciencias Visuales y Oftalmología, Instituto de Oftalmologia Fundacion Conde de Valenciana IAP, Mexico City, Mexico City, Mexico

The authors describe a very interesting and complete paper focused on the role of prevention in corneal blindness. 

 They mention the significance of epidemiological research and the attention that needs to be taken into account for a better understanding of risk factors, behavior and challenges of these conditions. Despite the challenges, conducting epidemiological research in developing countries is essential for appreciating and addressing the unique health issues faced by these populations. 

 The prevention strategies that they present can help organize and focus on the efforts that need to be done on each level. By focusing on early detection, addressing the root causes, and promoting public awareness, it is possible to reduce the incidence of corneal diseases and their associated blindness, thereby improving overall eye health and wellbeing in these populations. 

 Figures 2-3 legends are accidentally misplaced, figure 2 legend focuses on corneal scar after ocular trauma and the image and text are on ocular burns, the same is true regarding legend and figure 3, please address this.

Is the topic of the opinion article discussed accurately in the context of the current literature?

Yes

Are arguments sufficiently supported by evidence from the published literature?

Yes

Are all factual statements correct and adequately supported by citations?

Yes

Are the conclusions drawn balanced and justified on the basis of the presented arguments?

Yes

Reviewer Expertise:

Cornea, External Diseases, Refractive Surgery.

I confirm that I have read this submission and believe that I have an appropriate level of expertise to confirm that it is of an acceptable scientific standard, however I have significant reservations, as outlined above.
